# Epigenetic targeting of myeloid-derived suppressor cells: time to move into infectious diseases?

**DOI:** 10.3389/fimmu.2023.1247715

**Published:** 2023-08-23

**Authors:** Morten Frier Gjerstorff

**Affiliations:** ^1^ Department of Cancer and Inflammation Research, Institute of Molecular Medicine, University of Southern Denmark, Odense, Denmark; ^2^ Department of Oncology, Odense University Hospital, Odense, Denmark; ^3^ Academy of Geriatric Cancer Research (AgeCare), Odense University Hospital, Odense, Denmark

**Keywords:** cancer, MDSC (myeloid-derived suppressor cell), chronic infection, epigenetic inhibitor, inflammation

## Introduction

Evidence overwhelmingly suggests that myeloid-derived suppressor cells (MDSCs) play a crucial role in the pathogenesis of multiple types of infections, including tuberculosis, staphylococcal infections, viral hepatitis, immunodeficiency syndromes, chronic parasitic infections, and fungal disorders (1). Thus, there is pressing need to develop therapies that target these cells. DNMT inhibitors have demonstrated potential in cancer patients and preclinical cancer mouse models by effectively removing MDSCs. Given these observations, DNMT inhibitors should be considered as a potential treatment option for persistent infections in which MDSCs play a primary role, in addition to their role in cancer treatment.

## Targeting MDSCs with DNMT inhibitors in cancer treatment

DNA methyltransferase (DNMT) inhibitors, particularly decitabine and azacitidine, have been used for decades in the treatment of hematopoietic malignancies and are now being intensively investigated for their potential to treat solid cancers (2). These inhibitors target the enzymes responsible for adding methyl groups to DNA, which are known as DNA methyltransferases (DNMTs). DNA methylation is a crucial regulatory mechanism for controlling gene expression that is often disrupted during tumorigenesis. DNMT inhibitors, such as guadecitabine and decitabine, has been found to restore normal gene expression patterns in cancer cells, thereby reversing their oncogenic phenotypes. Besides their cancer cell-intrinsic effects, DNMT inhibitors have also shown promise in enhancing anti-tumor immune responses. They do so by counteracting the immune suppressive effect of MDSCs. MDSCs are immature, bone marrow-derived myeloid cells that are mobilized and recruited to the tumor microenvironment by inflammatory mediators produced within the tumor, where they play a crucial role in development of cancer immune resistance (3). MDSCs are divided into two subtypes: polymorphonuclear (PMN-) and mononuclear (M-) MDSCs. Although these subtypes are phenotypically and functionally distinct, they suppress T-cells by common mechanisms (3). These include secretion of immune suppressive molecules, such as nitric oxide (NO), reactive oxygen species (ROS), arginase, TGF-beta and IL-10, and induction of regulatory T-cells (Tregs). Encouragingly, multiple preclinical cancer mouse models have demonstrated that DNMT inhibitor treatment greatly reduces the recruitment of MDSCs to the tumor microenvironment and thereby enhance anti-tumor T-cell immune responses and improve the efficacy of cancer treatment (4, 5). This effect has been attributed to the ability of DNMT inhibitors to inhibit myelopoiesis in the bone marrow and MDSC expansion in the spleen (4). In addition, DNMT inhibitor treatment has been shown to change existing MDSC towards a less immune suppressive phenotype (5). While potently inhibiting tumor-associated excessive myeloid proliferation and systemic accumulation of MDSCs, DNMT inhibitors seem to have minimal effects on B- and T-cell populations (4). In fact, these drugs may increase activation and cytolytic activity of CD8^+^ T-cells (6).

## Targeting MDSCs in infectious diseases

**Figure 1:**
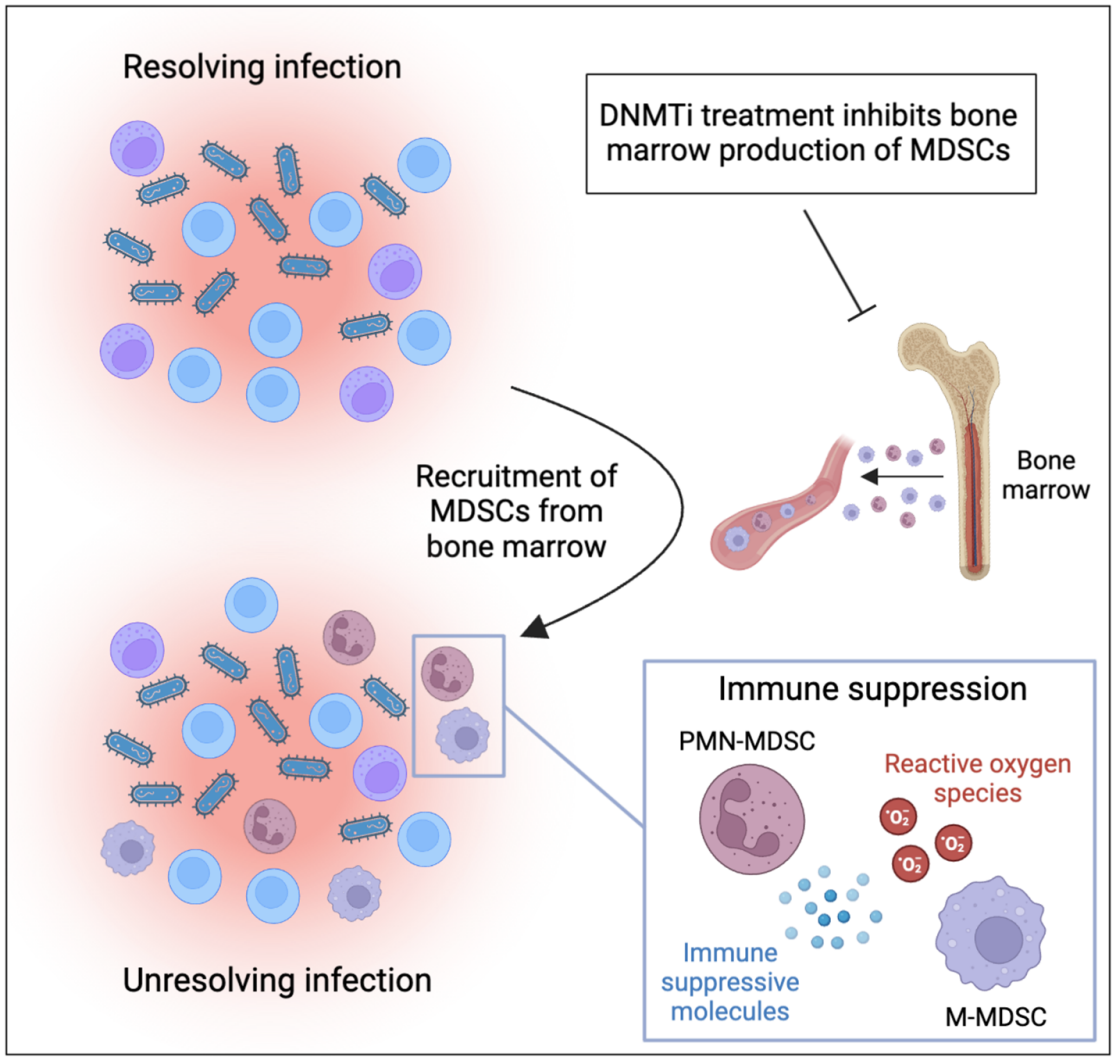
MDSCs contribute to persistence of infections and can be targeted by DNMT inhibitor treatment. Inflammatory cytokines produced during infection mobilize and recruit MDSCs to the site of infection. Recruited MDSCs suppress immune effector cells through production of reactive oxygen species and immune suppressive proteins. This ultimately suppresses immune responses against pathogens and contributes to unresolved inflammation and chronic infection. DNMT inhibitors have been demonstrated to inhibit the production of MDSCs in the bone marrow and should be considered for treatment of MDSC-driven infections (the figure was produced using BioRender).

MDSCs are also important regulators of the immune response against infectious diseases. They can be of benefit to the host by reducing the activity of T-cells, natural killer cells, and other immune effector cells that contribute to the immune response to protect the host from harmful overreactions of the immune system. On the other hand, excessive MDSC activity can prevent the immune system from mounting an effective response, which can ultimately favor pathogen persistence and contribute to unresolved chronic infections (**Figure 1**). For example, PMN-MDSCs have been found to expand in patients with sepsis, and their numbers correlate with disease severity (7, 8). In mouse models, anti-inflammatory MDSCs were found to be massively expanded during late stages of sepsis, contributing to bacterial growth, inhibiting T-cell function, and increasing mortality (9, 10). Mobilization of MDSC during sepsis seems to be mainly linked to infection with gram-positive bacteria (8).

MDSCs also play a role in the development of chronic bacterial infections (1). In tuberculosis patients, MDSCs inhibit T-cell responses and promote bacterial growth (11), while ablation of MDSCs in tuberculosis mouse models improves disease outcomes (12). Excessive accumulation of MDSCs in the lungs of *M. tuberculosis*-infected mice enhances disease severity, indicating that MDSCs provide a niche for *M. tuberculosis* survival within the infected lungs (12). Recruitment of MDSCs into infected tissues has also been reported in mice with Salmonella infection. In these mice, MDSCs were found to suppress T-cell responses by NO production (13). Likewise, MDSCs seem to play a crucial role in development of chronic *Staphylococcus aureus* infections, which is commonly associated with orthopedic biofilm formation and prolonged infections. MDSC were found to contribute to induce immune suppression and therapeutic resistance to this pathogen and depletion of MDSCs facilitated biofilm clearance (14).

Viral pathogens, such as HBV, HCV, HIV and EB virus, often develop chronic infections and MDSCs are increasingly being recognized as a contributor to immune suppression and viral persistence (1). For instance, MDSCs were expanded in the blood of patients with chronic HCV infection and these cells were shown to suppress T and NK cell anti-viral responses (15). Likewise, MDSCs may also play a negative role in HIV-induced immune deficiency syndrome, which is associated with T-cell dysfunction (15). In these patients, peripheral expansion of arginase-producing MDSCs correlates positively with viral load and drops upon antiviral therapy (15). On the other hand, there is evidence that MDSCs exert a protective role in chronic HBV infections, possibly by maintaining immune tolerance in HBV patients to avoid hepatic tissue damage (15).

The expansion of MDSCs has also been observed in infections caused by various types of protozoa and helminths (16). However, the role of MDSCs in parasitic infections remains a topic of debate. In certain parasitic infections, such as nematode infections, the increased presence of MDSCs has been shown to suppress T-cells, potentially contributing to the establishment of chronic infections (16). For example, in response to *H. pylogyrus bakeri* infection, MDSCs effectively inhibit CD4+ T-cell proliferation, leading to the suppression of CD4+ Th2 responses and the promotion of chronic infection (17). On the other hand, MDSCs seem to play a supportive role in the immune defense against several protozoans, including *Leishmania major* and *Trypanosoma cruzi* (18, 19). This beneficial effect is attributed to the production of nitric oxide (NO) by MDSCs, which aids in parasite clearance, while also potentially preventing excessive inflammation.

Similarly to parasitic infections, MDSCs also exhibit diverse roles in fungal infections. For instance, during infections caused by both *Aspergillus fumigatus* and *Candida albicans*, MDSCs are expanded. However, their impact differs, as they are protective against *Candida albicans* but not necessarily against *Aspergillus fumigatus* (20). Moreover, it has been observed that various species of *Candida* can induce different levels of MDSCs (21), indicating a species-specific response. Furthermore, in the context of *Cryptococcus neoformans* infection, MDSCs have been found to play an immune suppressive role, primarily mediated by the production of arginase (22). This indicates that the precise role of MDSCs in the defense against infections with parasites and fungi is highly influenced by the specific pathogen involved.

## DNMT inhibitors for treatment of infectious diseases

The growing recognition of MDSCs as key regulators of immune response in both cancer and infectious diseases highlights the potential of targeting these cells for therapeutic interventions. The ability of DNMT inhibitors to inhibit myelopoiesis, reduce MDSC recruitment, and transform existing MDSCs into a less immunosuppressive phenotype has shown great potential in preclinical cancer models. Given the lack of existing strategies to target MDSCs in infectious diseases, the use of DNMT inhibitors presents a promising avenue for intervention. While DNMT inhibitors have been extensively studied in the context of cancer, their potential application in infectious diseases remains unexplored. Further research is needed to investigate the efficacy of DNMT inhibitors in relevant infection models, assessing their ability to restore effective immunity and clear infections. In this context, they may be used as standalone agents or in combination with antibiotics. Pursuing these novel therapies can lead to significant progress in addressing the growing threat of untreatable infections.

## Discussion

Untreatable infections pose a significant threat to individuals and communities worldwide with millions of people dying each year and numbers are increasing. This makes the need for novel therapeutic strategies more critical than ever. Collectively, the above findings make a strong case for DNMT inhibitors as a powerful tool for relieving MDSC-controlled immune suppression and the use of DNMT inhibitors to target MDSCs in infectious diseases holds great promise and warrants further investigation. By modulating the immune response and reducing MDSC-mediated immunosuppression, DNMT inhibitors have the potential to become valuable therapeutic tools in the fight against untreatable infections. However, in some types of infections MDSCs contribute positively to the immune response by facilitating pathogen clearance and limiting inflammatory damage. Thus, further research is essential to fully understand the precise mechanisms by which MDSCs modulate immune responses in various types of infections and how this can be modulated with DNMT inhibitors.

## Author contributions

The author confirms being the sole contributor of this work and has approved it for publication.
